# Impact of socioeconomic and cardiovascular risk factors on the effect of genetic variants associated with NT-proBNP

**DOI:** 10.1038/s41598-022-19821-1

**Published:** 2022-09-16

**Authors:** Emanuel Matusch, Mirjam Frank, Kaffer Kara, Amir A. Mahabadi, Nico Dragano, Raimund Erbel, Karl-Heinz Jöckel, Börge Schmidt

**Affiliations:** 1 Institute for Medical Informatics, Biometry and Epidemiology, University of Duisburg-Essen, Hufelandstr. 55, 45122 Essen, Germany; 2 Department of Cardiology, Agaplesion Hospital Hagen, Hagen, Germany; 3 West German Heart and Vascular Center Essen, Department of Cardiology and Vascular Medicine, University Hospital Essen, University of Duisburg-Essen, Essen, Germany; 4 Institute of Medical Sociology, University Hospital Düsseldorf, Düsseldorf, Germany

**Keywords:** Risk factors, Epidemiology

## Abstract

N-terminal prohormone of brain natriuretic peptide (NT-proBNP) is an established biomarker for diagnosis of heart failure. The study aims to explore whether known cardiovascular risk factors, including education and income as indicators of socioeconomic position (SEP), may interact with the genetic effect of NT-proBNP-related single nucleotide polymorphisms (SNP) to influence plasma levels of NT-proBNP in a population-based study sample. Information on effect alleles of three SNPs previously reported to be related to NT-proBNP was combined individually for 4,520 participants of the Heinz Nixdorf Recall Study to calculate a genetic risk allele sum score (GRS_NT-proBNP_). Linear Regression models were used to examine the association of cardiovascular risk factors and GRS_NT-proBNP_ with log-transformed NT-proBNP levels, as well as cardiovascular risk factor by GRS_NT-proBNP_ interactions. The GRS_NT-proBNP_ was associated with NT-proBNP showing 1.13-fold (95% CI 1.10–1.16) higher plasma levels per additional effect allele. Interaction terms included in the regression models gave some indication for interaction of the GRS_NT-proBNP_ with the SEP indicator income as well as with C-reactive protein. In regression models stratified by income quartiles the strongest genetic effect was observed in the third income quartile showing 1.18-fold (95% CI 1.12–1.25) higher average NT-proBNP levels per additional allele compared to the lowest income quartile with 1.08-fold (95% CI 1.01–1.15) higher NT-proBNP levels. The results of the present study indicate that genetic effects of NT-proBNP increasing alleles are stronger in higher SEP groups. This may be due to a stronger influence of non-genetic cardiovascular risk on NT-proBNP in low SEP groups.

## Introduction

The biological inactive N-terminal prohormone of brain natriuretic peptide (NT-proBNP) and the biological active counterpart brain natriuretic peptide (BNP), initially discovered in brain tissue, are both products of the precursor peptide pro brain natriuretic peptide (proBNP) in cardiomyocytes. They are secreted under mechanical stress conditions (i.e. high ventricular volume) in a 1:1 ratio^1^. NT-proBNP is established in clinical routine for the diagnosis and evaluation of prognosis in patients with heart failure^2^. Different etiologies, for instance coronary artery disease, can cause heart failure, resulting in ventricular overload and thereby increasing NT-proBNP blood levels. Therefore, NT-proBNP has also been suggested for cardiovascular risk assessment^3^ and used to monitor pharmacological treatment success^4^. Moreover, NT-proBNP has been proposed as a biomarker for other diseases such as bronchopulmonary dysplasia after birth^5^, protein energy wasting in hemodialysis^6^ and cancer^7,8^.

However, NT-proBNP levels are influenced by risk factors, such as gender, age, antihypertensive medication (i.e., beta-blockers), renal insufficiency, obesity and testosterone levels^9–13^. Furthermore, indicators of socioeconomic position (SEP) (e.g., education, income, area deprivation) have been identified to be inversely associated with NT-proBNP levels^14,15^, yet there is limited research in this area. These clinical and social aspects have to be taken into account when interpreting NT-proBNP blood levels.

Recent genome-wide association studies have identified three single nucleotide polymorphism (SNP) alleles associated with increased NT-proBNP levels^16^. Like other common polygenetic risks for complex traits, main effects of the individual SNP alleles on NT-proBNP have been quite small^16^, indicating possible gene by environment interactions. It is assumed that SEP influences cardiovascular risk via its impact on the distribution of cardiovascular risk factors (CRF), meaning that SEP indicators can serve as proxy markers describing overall risk-related environments. It may then be hypothesized that SEP affects NT-proBNP by having an impact on the expression of NT-proBNP-related genes via its influence on CRFs^17,18^.

The aim of the present study was to explore whether a genetic risk allele sum score of NT-proBNP increasing alleles (GRS_NT-proBNP_) may interact with indicators of SEP (i.e., education, household income) and traditional CRFs (i.e., total cholesterol, high-density lipoprotein, low-density lipoprotein, triglycerides, C-reactive protein, serum glucose, HBA_1C_, body mass index, blood pressure, physical activity, diabetes mellitus, smoking status and coronary artery calcification) to influence plasma levels of NT-proBNP in a population-based study sample.

## Methods

### Study population

Baseline data of the Heinz Nixdorf Recall Study was used. Details of the study have been described elsewhere^19^. In brief, this ongoing population-based cohort study, starting in December 2000, included 4814 men and women from the German cities Essen, Bochum und Mülheim, aged 45 to 75 years. Participants were selected randomly by drawing a sample of the local resident registries, yielding a baseline response proportion of 55.8%^20^.

### Data acquisition

NT-proBNP was measured from frozen blood plasma, obtained from blood samples taken at study baseline, which were centrifuged and aliquoted for storage at -80 °C. Roche Modular E170 Assay (Roche Diagnostics, Mannheim, Germany) was used with the lowest analytical sensitivity reached at 5 pg/ml^21,22^_._

Education and income were used as indicators of SEP at study baseline. Education was defined using the International Standard Classification of Education (ISCED-97)^23^ and then categorized into three education groups (≤ 10 years, 11–13 years, ≥ 14 years of education) using the lowest education groups as reference for statistical analysis. Income was measured as the monthly household equivalent income in Euro calculated by dividing the total household net income by a weighting factor for each household member according to the OECD scale^24^. Calculations were performed using sex-specific income quartiles using the lowest quartile as reference.

Total cholesterol, high density lipoprotein (HDL) cholesterol and triglycerides in blood serum samples were determined using standard enzymatic assays. Low density lipoprotein (LDL) cholesterol was assessed via direct measurement and calculation using the Friedewald equation. Body Mass Index (BMI) was calculated by dividing measured baseline weight by squared baseline height. Blood glucose was determined by hexokinase reaction. High sensitivity C-reactive protein (CRP) and HbA_1C_ were measured by nephelometry (BN-II System, Dade-Behring Inc.). Blood pressure was assessed using the mean of the second and third of three measurements. Oscillometric blood pressure measurements were performed with an Omron HEM-705CP on sitting participants. Physical activity was assessed in computer-assisted face-to-face interviews and dichotomized as weekly physical exercise versus no weekly physical exercise. Smoking status was assessed in computer-assisted face-to-face interviews and dichotomized into current and former smokers versus never-smokers. Coronary artery calcification (CAC) was measured using EB-CT (electron beam computed tomography) with a GE Imatron scanner and the Agaston score was then computed as a measure of total CAC defined as the sum of the area (in mm^2^) of each detectable focus in the epicardial coronary system multiplied by its computed tomography density^25^. Glomerular filtration rate (GFR) was calculated using the modification of diet and renal disease formula (MDRD). Diabetes mellitus was defined as having blood glucose levels ≥ 200 mg/dl, fasting glucose > 125 mg/dl, or reported diabetes mellitus diagnosis or medication. Prevalent stroke and coronary heart disease at study baseline were assessed in computer-assisted face-to-face interviews.

### Genetic data

Selection of SNPs was based on the genome-wide association study of Johansson et al.^16^, in which three SNPs (rs198389 near *NPPB*; rs13107325 near *SLC39A8*; rs10858894 near *POC1B/GALNT4*) were robustly associated with NT-proBNP blood levels (Table 1).

**Table 1:** Overview of loci associated with increasing NT-proBNP levels selected according to Johansson et al.^16^.

SNP	Chromosome (position on GRCh37^†^)	Gene	Effective allele	Other allele	Effective allele frequency
rs198389	1 (11919271)	*NPPB*	G	A	0.43
rs13107325	4 (103188709)	*SLC39A8*	T	C	0.06
rs10858894^‡^	12 (89914261)	*POC1B/GALNT4*	T	C	0.77

^†^Genome Reference Consortium human build 37, version of a reference genome.

^‡^Proxy for rs11105306 in perfect linkage disequilibrium (R^2^ = 1.00).

Lymphocyte DNA of Heinz Nixdorf Recall Study participants was isolated from EDTA anti-coagulated venous blood using the Chemagic Magnetic Separation Module I (Chemagen, Baesweiler, Germany). Genotyping was performed using the Infinium Global Screening Array (GSA chip) by Illumina. For SNP rs11105306, originally reported by Johannson et al.^16^, the proxy SNP rs10858894 in perfect linkage disequilibrium (r^2^ = 1.0; 1000 Genomes CEU population) was used for statistical analysis. An unweighted genetic risk allele sum score (GRS_NT-proBNP_) was calculated for each participant by adding the number of NT-proBNP increasing effect alleles. No deviation from Hardy–Weinberg equilibrium (p > 0.001) was detected using an exact two-sided test.

### Statistical analyses

**Figure 1:**
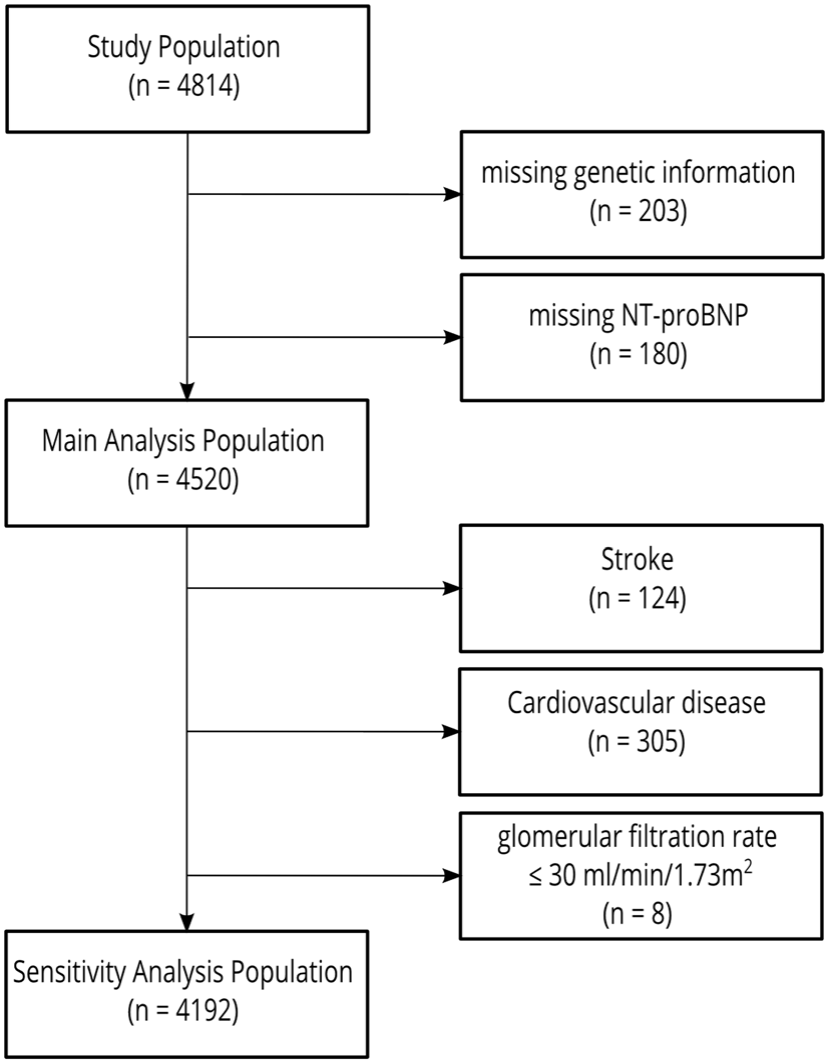
Flow-chart of participants of the Heinz Nixdorf Recall Study included in the analysis.

Genetic data and information on NT-proBNP were available for 4520 participants of the Heinz Nixdorf Recall Study (Fig. 1). Participants with missing data on SEP indicators and other CRFs were only excluded from the respective analyses.

To normalize the distribution of NT-proBNP a log-transformation was applied. Effect size estimates were presented back-transformed (exp[β]). Main effects of the three SNP effect alleles, the GRS_NT-proBNP_, SEP indicators and CRFs on NT-proBNP were assessed by fitting age- and sex-adjusted linear regression models separately for each variable. Interaction was explored by additionally including GRS_NT-proBNP_*SEP/CRF interaction terms and SEP indicator/CRF main effects in age- and sex-adjusted linear regression models separately for each SEP indicators and CRF (i.e., log(NT-proBNP) = age + sex + GRS_NT-proBNP_ + SEP/CRF + GRS_NT-proBNP_*SEP/CRF). SEP indicators were included as dummy variables in the regression models with the first income quartile and lowest education group as reference category, respectively. The GRS_NT-proBNP_ effect was also calculated stratified by groups of SEP indicators and those CRFs showing indication for interaction. Sensitivity analysis was performed by including only participants without stroke and coronary artery disease at baseline and with a GFR of > 30 ml/min/1.73m^2^. All statistical analyses were performed using the statistical software package R, version 3.5.3^26^.

### Ethics approval and consent to participate

Written informed consent was retrieved from all participants and the study was approved by the ethics committee of the University Duisburg-Essen. The study complies with the quality management system DIN ISO 9001:2000. The study was conducted according to the guidelines and recommendations for ensuring Good Epidemiological Practice (https://www.dgepi.de/assets/Leitlinien-und-Empfehlungen/Recommendations-for-good-Epidemiologic-Practice.pdf).

## Results

Median NT-proBNP plasma levels of 2263 male and 2257 female participants, mean aged 60 years, were 70.0 pg/ml, with women having higher median NT-proBNP levels (86.0 pg/ml, IQR 52.0–147.0) than men (54.0 pg/ml, IQR 31.0–109.0) in the study population (Table 2). Indicators of SEP showed sex-differences with women having a lower median income compared to men (1313€ vs. 1520€) and on average less years of education. The mean number of GRS_NT-proBNP_ effect alleles did not differ between women and men (2.6 ± 1.0 vs. 2.5 ± 1.0) in the study population.

**Table 2:** Description of analysis population.

Variable	All (n = 4520)	Men (n = 2263)	Women (n = 2257)
Age^‡^ (years)	59.6 ± 7.8	59.7 ± 7.8	59.6 ± 7.8
Genetic Risk Score_NT-proBNP_^‡^	2.5 ± 1.0	2.5 ± 1.0	2.6 ± 1.0
NT-proBNP^†^ (pg/ml_	70.0 (39.0–132.0)	54.0 (31.0–109.0)	86.0 (52.0–147.0)
monthly Income^†^ (€) (n_miss_ = 284)	1449 (1108–1875)	1520 (1108–2073)	1313 (0937–1875)
**Education groups (n**_**miss**_** = 13)**			
≤ 10 years	517 (11.5%)	111 (4.9%)	406 (18.0%)
11–13 years	2494 (55.3%)	1073 (47.6%)	1421 (63.0%)
≥ 14 years	1496 (33.2%)	1069 (47.5%)	427 (19.0%)
C-reactive protein^†^ (mg/l) (n_miss_ = 11)	0.2 (0.1–0.3)	0.2 (0.2–0.3)	0.2 (0.1–0.3)
Serum Glucose^†^ (mg/dl) (n_miss_ = 4)	105.0 (98.0–115.0)	108.0 (101.0–119.0)	103.0 (96.0–111.0)
Total Cholesterol^‡^ (mg/dl) (nmiss = 1)	229.1 ± 39.2	225.0 ± 39.1	233.3 ± 39.4
High density lipoprotein cholesterol^‡^ (mg/dl) (n_miss_ = 2)	58.1 ± 17.2	51.1 ± 17.2	65.17 ± 17
Low density lipoprotein cholesterol^‡^ (mg/dL) (n_miss_ = 14)	145.6 ± 36.1	145.5 ± 36.1	145.7 ± 36.8
Triglycerides^†^ (mg/dL) (n_miss_ = 4)	124.0 (90.0–178.0)	137.5(96.0–200.0)	114.0(85.0–159.0)
HBA_1c_^†^ (%) (n_miss_ = 34)	5.4 (5.1–5.7)	5.4 (5.1–5.8)	5.3 (5–5.7)
Body mass index^‡^ (kg/m^2^) (n_miss_ = 26)	27.9 ± 4.6	28.2 ± 4.6	27.6 ± 5.2
No physical activity (n_miss_ = 1)	2072 (45.8%)	1052 (46.5%)	1237 (54.8%)
Smoking (n_miss_ = 7)	2626 (58.2%)	1622 (71.8%)	1004 (44.5%)
Systolic blood pressure^‡^ (mmHg) (n_miss_ = 12)	133.2 ± 20.8	138.1 ± 20.8	128.2 ± 21.0
Diastolic blood pressure^‡^ (mmHg) (n_miss_ = 11)	81.5 ± 10.8	84.0 ± 10.8	78.9 ± 10.6
Diabetes mellitus	607 (13.4%)	390 (17.2%)	217 (9.6%)
Coronary artery calcification (n_miss_ = 191)	18.6 (0.0–166.6)	77.1 (6.7–360.5)	1.80 (0.0–43.38)
Stroke at baseline (n_miss_ = 13)	124 (2.8%)	76 (3.4%)	48 (2.1%)
coronary artery disease at baseline (n_miss_ = 10)	305 (6.8%)	242 (10.7%)	63 (2.8%)
GFR‡ [ml/min] (n_miss_ = 5)	79.5 ± 18.4	83.0 ± 18.4	76.0 ± 18.5

^‡^Mean ± standard deviation.

^†^Median (first quartile − third quartile).

n_miss_ = missing value.

Per additional effect allele of the GRS_NT-proBNP_ the average NT-proBNP level was 1.13-fold (95% CI 1.10–1.16) higher (Table 3). Each effect allele of the three selected SNPs showed directional consistent effects as previously reported. The strongest effect was observed for rs13107325 with an exp(β) of 1.21 (95% CI 1.12–1.30) per additional effect allele. Using the first income quartile as reference, NT-proBNP levels decreased with increasing income (Table 3). In the fourth quartile the effect strength of income showed an exp(β) of 0.90 (95% CI 0.83–0.97) compared to the first income quartile. No association between education and NT-proBNP was observed. Positive associations with NT-proBNP were observed for CRP, systolic blood pressure, no physical activity, diabetes mellitus, smoking and coronary artery calcification. Negative associations were observed for total cholesterol and LDL.

**Table 3:** Exp(β) and 95% confidence intervals (95% CI) for the association of genetic, socioeconomic and cardiovascular risk factors with NT-proBNP in separate linear regression models, adjusted for sex and age (lowest income/education group as reference).

Variable	n	Exp(β)	95% confidence interval	p-value
Genetic risk score_NT-proBNP_‡	4520	1.13	1.10–1.16	3.8 × 10^–21^
rs198389	4520	1.16	1.12–1.20	2.0 × 10^–15^
rs13107325	4520	1.21	1.12–1.30	8.7 × 10^–7^
rs11105306	4520	1.08	1.04–1.13	3.5 × 10^–4^
**Income quartiles**	4236			
2. quartile		1.00	0.93–1.08	0.99
3. quartile		0.98	0.90–1.06	0.61
4. quartile		0.90	0.83–0.97	0.01
**Education groups**	4507			
11–13 years		1.04	0.96–1.13	0.35
≥ 14 years		0.98	0.89–1.07	0.64
C-reactive protein (mg/dl)	4509	1.05	1.02–1.08	2.5 × 10^–4^
Serum Glucose^†^ (mg/dl)	4516	1.00	0.98–1.02	0.94
Total Cholesterol^‡^ (mg/dl)	4519	0.88	0.86–0.90	4.9 × 10^–23^
High density lipoprotein cholesterol^‡^ (mg/dl)	4518	1.00	0.98–1.03	0.83
Low density lipoprotein cholesterol^‡^ (mg/dl)	4506	0.87	0.85–0.89	2.7 × 10^–26^
Triglycerides^†^ (mg/dl)	4516	0.99	0.97–1.01	0.41
HBA_1C_^†^ (%)	4486	1.00	0.97–1.04	0.77
Body Mass Index^‡^ [kg/m^2^]	4494	0.99	0.97–1.02	0.46
Systolic blood pressure^‡^ (mmHg)	4508	1.07	1.04–1.10	3.1 × 10^–6^
Diastolic blood pressure^‡^ (mmHg)	4509	1.00	0.97–1.03	0.92
No physical activity	4519	1.07	1.02–1.13	6.1 × 10^–3^
Diabetes mellitus	4520	1.04	0.97–1.13	0.26
Smoking	4513	1.08	1.03 – 1.14	3.8 × 10^–3^
Coronary artery calcification†	4329	1.05	1.04– 1.06	3.0^‡^ 10^–40^

^‡^Exp(β) per standard deviation.

^†^Exp(β) per interquartile range.

Results of the interaction analysis including GRS_NT-proBNP_ by SEP/CRF interaction terms gave some indication for a positive interaction between GRS_NT-proBNP_ and income for comparing the first and the third income quartile (exp(β_GRSxIncome_) = 1.10, 95% CI 1.01–1.19) as well as between GRS_NT-proBNP_ and CRP (exp(β_GRSxCRP_) = 1.03, 95% CI 1.01–1.06) on NT-proBNP (Table 4). However, after including income and the related income interaction terms in the regression model containing CRP and the GRS_NT-proBNP_ by CRP interaction term, the effect size estimate of the observed GRS_NT-proBNP_ by CRP interaction term dropped to 1.00 (95% CI 0.96–1.04), while the effect size for GRS_NT-proBNP_ by income interaction terms remained virtually the same.

**Table 4:** Exp(β_interaction_) and 95% confidence intervals (95% CI) of the interaction term for the interaction of socioeconomic and cardiovascular risk factors by the NT-proBNP-related genetic risk allele sum score on NT-proBNP in linear regression models separately for each socioeconomic and cardiovascular risk factor, adjusted for sex and age (lowest income/education group as reference).

Variable	n	Exp (β_interaction_)	95% confidence interval	p-value
**Income quartiles**	4236			
2. quartile		1.04	0.96–1.13	0.29
3. quartile		1.10	1.01–1.19	0.03
4. quartile		1.06	0.98–1.15	0.17
**Education groups**	4507			
11–13 years		1.02	0.94–1.10	0.70
≥ 14 years		1.03	0.95–1.13	0.45
C-reactive protein (mg/dl)	4509	1.03	1.01–1.06	0.02
Serum glucose^†^ (mg/dl)	4516	1.00	0.99–1.02	0.82
Total cholesterol^‡^ (mg/dl)	4519	0.99	0.96–1.01	0.26
High density lipoprotein cholesterol^‡^ (mg/dl)	4518	1.00	0.97–1.02	0.84
Low density lipoprotein cholesterol^‡^ (mg/dl)	4506	1.00	0.97–1.02	0.82
Triglycerides^†^ (mg/dl)	4516	0.99	0.96–1.01	0.21
HBA_1C_^†^ (%)	4486	0.99	0.96–1.02	0.70
Body Mass Index^‡^ (kg/m^2^)	4494	0.99	0.96–1.01	0.24
Systolic blood pressure^‡^ (mmHg)	4508	0.99	0.96–1.01	0.36
Diastolic blood pressure^‡^ (mmHg)	4509	1.00	0.97–1.02	0.80
No physical activity	4519	1.00	0.95–1.05	0.95
Diabetes mellitus	4520	0.96	0.89–1.04	0.32
Smoking	4513	0.98	0.93–1.04	0.53
Coronary artery calcification^†^	4329	0.99	0.99–1.00	0.07

^‡^Exp(β) per standard deviation.

^†^Exp(β) per interquartile range.

**Figure 2:**
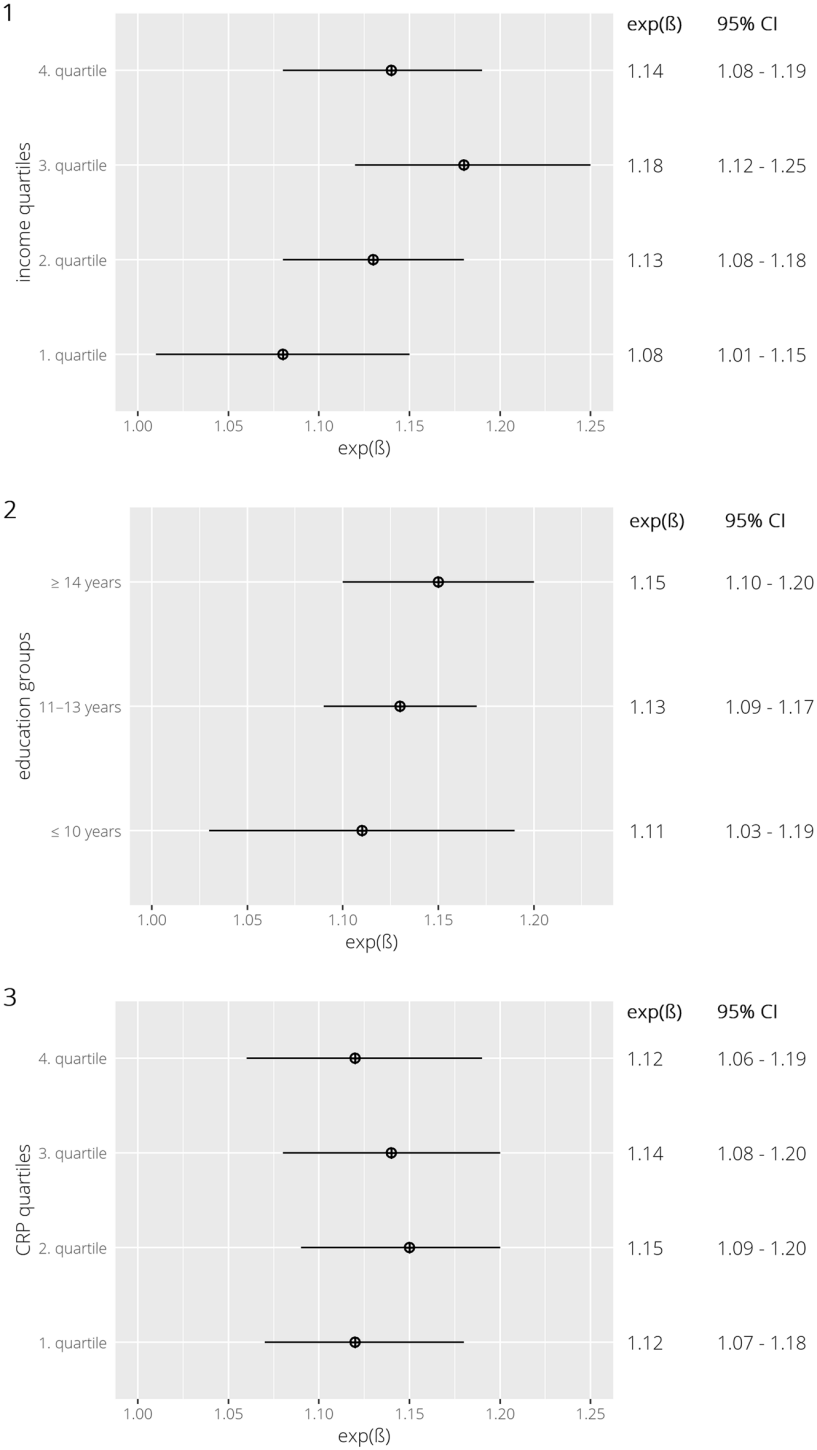
Exp(β) and 95% confidence intervals (95% CI) for the effect of the genetic risk allele sum score on NT-proBNP per additional effect allele stratified by income quartiles (1), education groups (2) and C-reactive protein quartiles (3).

The effect of the GRS_NT-proBNP_ stratified by income quartiles showed an increase of the genetic effect on NT-proBNP with increasing income quartiles, except for the fourth income quartile (Fig. 2). The strongest effect of GRS_NT-proBNP_ was observed in the third income quartile showing an exp(β) of 1.18 (95% CI 1.12–1.25) compared to an exp(β) of 1.08 (95% CI 1.01–1.15) in the first income quartile. The effect of the GRS_NT-proBNP_ stratified by education groups also showed an increase in the strength of the genetic effect with increasing years of education (Fig. 2). However, compared to the stratified analysis using income quartiles, differences in the genetic effect size estimates between education groups were less strong. After calculating the effect of the GRS_NT-proBNP_ on NT-proBNP stratified by CRP quartiles, only small differences in effect size were observed lacking a general trend of effects across CRP strata (Fig. 2). However, the effect size estimate of rs13107325 effect alleles on NT-proBNP show an increasing trend across CRP quartiles in the single SNP analyses (Fig. S1). Single SNP effects on NT-proBNP stratified by income and education showed that for income the effect of all three SNPs was again strongest in the third quartile with the strongest effect across all SNPs for rs13107325 (exp(β) of 1.35 [95% CI 1.14–1.60]), while for education again smaller differences in the genetic effect were observed between groups compared to income quartiles (Fig. S1).

After including only participants without stroke, coronary artery disease and a GFR > 30 ml/min/1.73 m^2^ in a sensitivity analysis of the main results, only small changes in effect size estimates were observed for the association of SEP indicators and CRFs with NT-proBNP (Table 5). Effect size estimates for the GRS_NT-proBNP_ by SEP and GRS_NT-proBNP_ by CRP interaction effect were slightly smaller compared to the main analyses population (Table 6). In a single SNP analysis, the strongest indication for interaction was observed for rs13107325 interaction with CRP (Table S1).

**Table 5:** Exp(β) and 95% confidence intervals (95% CI) for the association of genetic, socioeconomic and cardiovascular risk factors with NT-proBNP in separate linear regression models, adjusted for sex and age, including only participants with no stroke, no coronary artery disease and glomerular filtration rate > 30 ml/min/1.73m^2^ at baseline (lowest income/education group as reference).

Variable	n	exp(β)	95% Confidence Interval	p-value
Genetic risk score_NT-proBNP_	4192	1.14	1.11–1.17	8.9 × 10^–21^
rs198389	4192	1.17	1.13–1.22	2 × 10^–18^
rs13107325	4192	1.20	1.11–1.29	1.7 × 10^–6^
rs11105306	4192	1.09	1.05–1.14	3.8 × 10^–5^
**Income quartiles**	3927			
2. quartile		1.02	0.95–1.10	0.54
3. quartile		0.98	0.90–1.06	0.56
4. quartile		0.92	0.85–1.00	0.04
**Education groups**				
11–13 years		1.02	0.94–1.11	0.69
≥ 14 years		0.96	0.88–1.06	0.41
C-reactive protein (mg/dl)	4181	1.03	1.01–1.06	0.01
Serum glucose^†^ (mg/dl)	4190	0.99	0.97–1.00	0.13
Total cholesterol^‡^ (mg/dl)	4192	0.91	0.89–0.93	1.5*10^–13^
High density lipoprotein cholesterol^‡^ (mg/dl)	4191	1.02	0.99–1.05	0.11
Low density lipoprotein cholesterol^‡^ (mg/dl)	4180	0.90	0.87–0.92	6.7*10^–18^
Triglycerides^†^ (mg/dl)	4189	0.99	0.97–1.02	0.66
HBA_1C_^†^ (%)	4159	0.98	0.95–1.01	0.23
Body Mass Index^‡^ (kg/m^2^)	4172	0.99	0.97–1.02	0.53
Systolic blood pressure^‡^ (mmHg)	4184	1.09	1.06–1.12	9.2*10^–11^
Diastolic blood pressure^‡^ (mmHg)	4185	1.03	1.00–1.06	0.03
No physical activity	4191	1.06	1.01–1.12	0.02
Diabetes mellitus	4192	0.96	0.89–1.04	0.33
Smoking	4188	1.05	1.00–1.11	0.06
Coronary artery calcification^†^	4021	1.03	1.02–1.04	3.0 × 10^–17^

^‡^Exp(β*standard deviation(β)).

^†^Exp(β*IQR(β)).

**Table 6:** Exp(β_interaction_) and 95% confidence intervals (95% CI) of the interaction term for the interaction of socioeconomic and cardiovascular risk factors by the NT-proBNP-related genetic risk allele sum score on NT-proBNP in separate linear regression models, separately for each socioeconomic and cardiovascular risk factor, adjusted for sex and age, including only participants with no stroke, no coronary artery disease and glomerular filtration rate > 30 ml/min/1.73 m^2^ at baseline (lowest income/education group as reference).

Variable × genetic effect allele sum score_NT-proBNP_	n	Exp (β_interaction_)	95% confidence interval	p-value
**Income quartiles**	3927			
2. quartile		1.03	0.95–1.11	0.45
3. quartile		1.06	0.98–1.15	0.18
4. quartile		1.05	0.97–1.13	0.26
**Education groups**	4182			
11–13 years		1.01	0.93–1.09	0.84
≥ 14 years		1.01	0.93–1.10	0.75
C-reactive protein (mg/dl)	4181	1.02	0.99–1.04	0.22
Serum glucose^†^ (mg/dl)	4190	1.01	0.99–1.02	0.46
Total cholesterol^‡^ (mg/dl)	4192	0.99	0.96–1.01	0.37
High density lipoprotein cholesterol^‡^ (mg/dl)	4191	1.00	0.98–1.03	0.86
Low density lipoprotein cholesterol^‡^ (mg/dl)	4180	1.00	0.97–1.02	0.82
Triglycerides^†^ (mg/dl)	4189	0.99	0.97–1.01	0.25
HBA_1C_ (%)	4159	1.01	0.98–1.04	0.42
Body Mass Index^‡^ (kg/m^2^)	4172	0.99	0.96–1.01	0.37
Systolic blood pressure^‡^ (mmHg)	4184	0.98	0.96–1.01	0.16
Diastolic blood pressure^‡^ (mmHg)	4185	0.99	0.96–1.01	0.32
No physical activity	4191	1.02	0.97–1.07	0.49
Diabetes mellitus	4192	0.98	0.90–1.05	0.53
Smoking	4188	0.99	0.95–1.05	0.83
Coronary artery calcification^†^	4021	1.00	1.00–1.01	0.36

^‡^Exp(β) per standard deviation.

^†^Exp(β) per interquartile range.

## Discussion

The present study aimed to explore the impact of established CRFs and SEP indicators on the effect of a GRS_NT-proBNP_ in a population-based cohort, including three SNPs previously reported to be associated with NT-proBNP blood levels. The observed association between the GRS_NT-proBNP_ and NT-proBNP levels was directionally consistent, replicating the findings of Johansson et al.^16^, as the cumulative and individual effects of the selected SNPs on NT-proBNP were demonstrated in an independent study sample with rs13107325 reaching genome-wide significance. However, there was heterogeneity of the effect strength across groups of different household income used as SEP indicator. Groups with higher SEP experienced stronger genetic effects compared to groups with lower SEP. Indication for a positive GRS_NT-proBNP_ by CRP interaction was also observed suggesting stronger genetic effects with increasing CRP. However, after adjustment for confounding by income, the GRS_NT-proBNP_ by CRP interaction disappeared, while the GRS_NT-proBNP_ by income interaction was still present. Overall, results were not strongly affected by prevalent cardiovascular disease or GFR.

In a previous study, social inequality in NT-proBNP levels has already been reported^15^, which may be explained by a higher prevalence of NT-proBNP-related health risks in lower SEP groups. In a twin study by Johnson and Krueger^17,27^ it has been observed that the genetic influence on overall measures of physical health decreased with increasing income. This may be interpreted as an impact of non-genetic risk factors associated with SEP on the expression of adverse effects of health-related genetic variants. However, the direction of this effect modification is not in line with the results of the study presented here and stronger genetic effects on NT-proBNP in groups of high SEP have to be explained by a different mechanism. One possible explanation may be that the effect of the selected SNPs on NT-proBNP becomes negligible in the presence of much stronger effects of non-genetic risk factors associated with SEP, while in high SEP groups with lower rates of health risks a greater direct influence of genetic factors on NT-proBNP can be observed. As proposed in previous studies, cardiovascular diseases are more prevalent in lower SEP groups^28^. It is hypothesized that absolute NT-proBNP levels may compensate cardiovascular risk due to a mechanism that regulates cardiac preload, as NT-proBNP reduces blood pressure and preload via natriuresis^29,30^. Smaller genetic effects on NT-proBNP in groups of low SEP might be a result of CRFs inducing a regulatory NT-proBNP increase that outweighs the genetic effect on NT-proBNP. Thus, groups of high SEP may experience stronger genetic effects on NT-proBNP, because cardiovascular risks are less prevalent in those groups. Moreover, it may be speculated that people with genetically incremented NT-proBNP levels might experience a protective factor against certain cardiovascular diseases, for instance heart failure^31^.

Strengths of the current study are the population-based design and the use of a range of established CRFs for analysis. However, the cross-sectional study design is a limitation as individuals who already experienced a fatal cardiovascular event could not be included in the analyses population. Another limitation is that the study results may not be transferable to other ethnic groups as participants are mainly of Caucasian origin.

In conclusion, the results of the present study indicate that genetic effects of NT-proBNP increasing alleles are stronger in higher SEP groups. This may be due to a stronger influence of non-genetic cardiovascular risk on NT-proBNP in low SEP groups. Any interactions between NT-proBNP genetic effects and traditional CRFs may be confounded by SEP interactions.

## Supplementary Information

Supplementary Information.

## Data Availability

Due to data security reasons (i.e., data contain potentially participant identifying information), the Heinz Nixdorf Recall Study does not allow sharing data as a public use file. However, others can access the data used upon request, which is the same way the authors of the present paper obtained the data. Data requests can be addressed to: recall@uk-essen.de.
