# Supplementary material for: Impact of socioeconomic and cardiovascular risk factors on the effect of genetic variants associated with NT-proBNP
Source: Sci Rep. 2022 Sep 16;12:15560. doi: 10.1038/s41598-022-19821-1 (PMC9481588; doi:10.1038/s41598-022-19821-1)
Supplement: Supplementary file 1 — Supplementary Information. [file 41598_2022_19821_MOESM1_ESM.pdf]

Supplement

Table S1: Exp(β) and 95% confidence intervals (95% CI) for the interaction of socioeconomic indicators and C-reactive protein with NT-proBNP-related genetic risk alleles of single SNPs on NT-proBNP in separate linear regression models, adjusted for sex and age (lowest income/education group as reference).

Variable	n	$\exp(\beta_{interaction})$	95% Confidence interval	p-value
rs198389				
Income quartile 2	4236	1.04	0.93 – 1.16	0.47
Income quartile 3		1.10	0.98 – 1.23	0.11
Income quartile 4		1.05	0.94 – 1.18	0.36
Education group 11 – 13 years	4507	1.02	0.91 – 1.15	0.74
Education group ≥ 14 years		1.04	0.92 – 1.17	0.56
C-reactive protein	4509	1.03	0.99 – 1.07	0.11
rs13107325				
Income quartile 2	4236	0.99	0.79 – 1.23	0.91
Income quartile 3		1.14	0.89 – 1.45	0.29
Income quartile 4		1.06	0.83 – 1.34	0.65
Education group 11 – 13 years	4507	1.03	0.81 – 1.31	0.80
Education group ≥ 14 years		1.00	0.77 – 1.29	0.97
C-reactive protein	4509	1.31	1.07 – 1.59	0.01
rs11105306				
Income quartile 2	4236	1.07	0.94 – 1.22	0.30
Income quartile 3		1.09	0.95 – 1.25	0.22
Income quartile 4		1.09	0.95 – 1.25	0.22
Education group 11 – 13 years	4507	0.99	0.87 – 1.14	0.90
Education group ≥ 14 years		1.05	0.91 – 1.22	0.51
C-reactive protein	4509	1.07	1.01 – 1.13	0.02

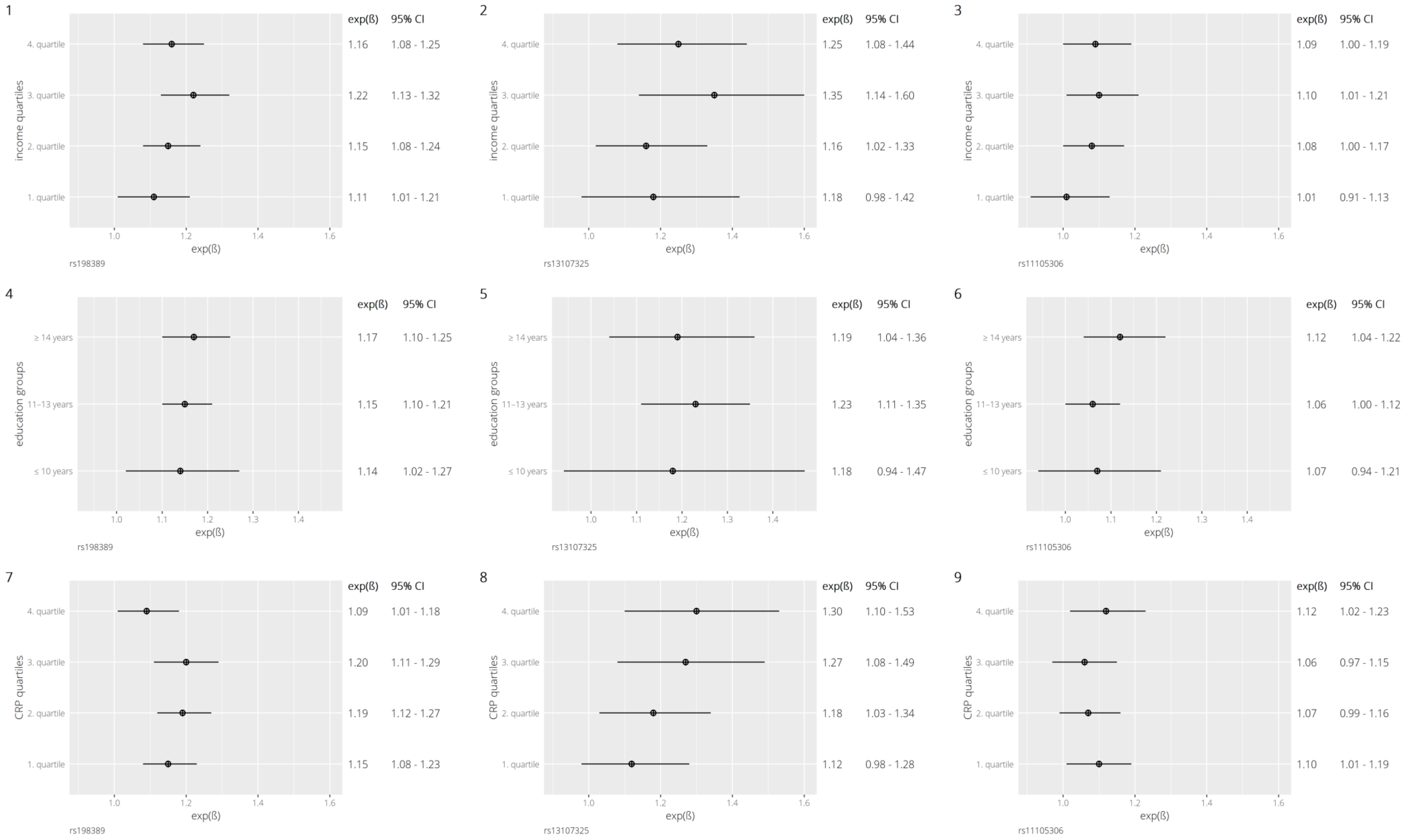

Figure S1. Exp(β) and 95% confidence intervals (95% CI) for the effect of single SNP effects on NT-proBNP per additional effect allele stratified by income quartiles (1-3), education groups (4-6) and C-reactive protein quartiles (7-9).
